# Ideal hourglass nodal loop state in the monolayer lithium hydrosulfide

**DOI:** 10.3389/fchem.2024.1500989

**Published:** 2024-12-02

**Authors:** Fang Fang, Yanwei Yu, Li Zhang, Yang Li

**Affiliations:** ^1^ The Engineering and Technology Research Center of Myocardial Prevention and Rehabilitation, The Fourth Medical College of Harbin Medical University, Harbin, China; ^2^ TOF-PET/CT/MR Center, The Fourth Medical College of Harbin Medical University, Harbin, China; ^3^ College of Mechanics, Changchun Institute of Technology, Changchun, China

**Keywords:** nodal loop state, topological state, topological material, hourglass dispersion, two dimensional material

## Abstract

In recent years, the exploration of topological states within two-dimensional materials has emerged as a compelling focus, complementing their three-dimensional counterparts. Through theoretical calculations, we unveil the exceptional topological state in the monolayer lithium hydrosulfide, where an ideal hourglass nodal loop is identified. Notably, this nodal loop is characterized by only four bands, representing the simplest configuration for realizing hourglass dispersion. We provide detailed symmetry arguments alongside model calculations to elucidate the formation mechanism of the nodal loop and its corresponding hourglass dispersion. Moreover, the associated edge states are not only well-separated from the bulk band projection but also persist consistently throughout the Brillouin zone. Due to the lightweight constitutive elements of this material, both the hourglass dispersion and the edge states remain robust even in the presence of spin-orbit coupling. To enhance its practical applicability, we have evaluated various mechanical parameters, analyzing their anisotropic behaviors. Furthermore, we examined the material’s response to strain conditions under both compressive and tensile stress, uncovering distinct variations in energy, size, and the hourglass dispersion of the nodal loop. Overall, the hourglass nodal loop state explored in this study, along with the proposed material candidate, provides a strong foundation for future experimental investigations. This research potentially paves the way for significant advancements within this emerging field.

## Introduction

Significant progress has been achieved in the exploration of topological states within the fields of condensed matter physics and solid-state materials (Xiao and Yan, 2021; Zhang et al., 2019a; Tang et al., 2019a; Tang et al., 2019b), particularly following the development of topological band theory (Bansil et al., 2016; Chiu et al., 2016; Cooper et al., 2019; Hasan and Kane, 2010; Qi and Zhang, 2011; Wang et al., 2024; Wang et al., 2023; Gong et al., 2024; Singh et al., 2023; Li et al., 2023a; Wang et al., 2022; Yang et al., 2024). This theoretical framework is vital for elucidating the characteristics of topological states in crystalline materials, linking them to structural symmetry operations and the limitations dictated by band topology. As research has evolved, the focus of investigation has expanded beyond just topological insulators to include a broader array of systems, encompassing topological semimetals (Soluyanov et al., 2015; Burkov, 2016; Yan and Felser, 2017; Gao et al., 2019; Burkov, 2018; Schoop et al., 2018; Weng et al., 2016; Bernevig et al., 2018; Hirayama et al., 2018; Burkov et al., 2011; Armitage et al., 2018), as well as topological phonons (Yang et al., 2019; Long et al., 2020; Wang et al., 2020; Xia et al., 2019; Liu et al., 2022), photons (Liu et al., 2021; Yang et al., 2018; Pan et al., 2023; Lin et al., 2023; Hu et al., 2022; Deng et al., 2022), and magnons (Li et al., 2017; Nie et al., 2020; Zhu et al., 2021; Corticelli et al., 2022; Moghaddam et al., 2022; Gordon et al., 2021; He et al., 2021). Depending on their unique configurations, topological quasiparticles in solid-state systems can exhibit diverse pseudospin structures, topological charges, and dispersion types, along with various topological manifolds. A primary hallmark of topological properties is the manifestation of nontrivial surface states, which serve as the defining feature of topological properties and are frequently employed in both theoretical calculations and experimental analyses to ascertain corresponding topological states (Neupane et al., 2016; Xu et al., 2015; Zhang et al., 2019b; Xiao et al., 2020; Yu et al., 2017; Zhang et al., 2020; Takane et al., 2019; Yang et al., 2023). For example, the Fermi arc spectrum emerges from the crossing points associated with topological nodal points, while drumhead surface states arise between crossing lines or within crossing loops for topological nodal lines or loops. These nontrivial surface states are fundamental to the topological characteristics associated with crystal space group symmetries and present exciting prospects for the development of new quantum devices and applications.

During recent years, the exploration of topological states in two-dimensional materials has rapidly ascended to the forefront of scientific inquiry, augmenting the conventional studies of their three-dimensional counterparts (Chen et al., 2024; Zhong et al., 2024; Xie et al., 2023; Li et al., 2023b; Liu et al., 2023; Zhong et al., 2023; Yu et al., 2023; Guo et al., 2023a; Guo et al., 2023b; Zhang et al., 2023). This burgeoning interest is not only driven by the novel physics these systems offer, but also by their potential to revolutionize current technologies. Two-dimensional materials, with their unique properties, have opened up a plethora of possibilities for practical applications (Miró et al., 2014; Feng et al., 2021; Zhou, 2020; Jiang and Mi, 2023). Their thin, planar nature facilitates structural integration, allowing for a seamless incorporation into a variety of systems. This adaptability makes two-dimensional materials highly compatible with existing technologies, thereby reducing the barriers to their practical use. Moreover, the intrinsic planarity of these materials simplifies their incorporation into devices, providing a straightforward pathway for the development of advanced applications. This ease of integration, combined with the novel properties these materials exhibit, positions two-dimensional materials as a promising frontier in the quest for next-generation technologies. This positions them as promising candidates for various applications. Similar to their bulk counterparts, two-dimensional materials exhibit analogous topological behaviors. Specifically, various topological states can be distinguished based on characteristics such as band degeneracy overlap, band dispersion conditions, and the arrangement of band crossings. However, a key distinction in two-dimensional materials arises from their reduced dimensionality, which typically leads to a transition from topological surface states to edge states. While substantial advancements have been made in this area, the investigation of topological states remains an active and rapidly evolving field, continuously presenting new challenges and opportunities. Notably, the search for ideal topological states in two-dimensional systems is urgent, as the range of available material candidates is still considerably limited in comparison to three-dimensional materials. This underscores the critical need for further discovery and exploration of new materials, particularly those that exhibit ideal topological states and straightforward topological configurations.

In this study, we identify monolayer lithium hydrosulfide as a highly stable candidate material with remarkable topological properties. Through theoretical analyses and effective model calculations, we demonstrate that this two-dimensional compound exhibits an ideal hourglass nodal loop state within its top valence bands. This unique nodal loop is characterized by only four bands, free from interference from other bands, representing the simplest configuration for achieving hourglass dispersion. We utilize symmetry arguments to explain the formation mechanism of the nodal loop and its hourglass dispersion. Additionally, three-dimensional band surface scans provide further validation of the hourglass crossings throughout the entire nodal loop. Notably, this loop displays a flat profile in terms of both energy dispersion and spatial distribution, occupying a substantial area in space, which is highly advantageous for experimental characterization and practical applications. Furthermore, we find that the calculated edge states along the (100) direction are well-separated from the bulk band projection, persisting throughout the Brillouin zone. Importantly, due to the lightweight constituent elements, both the hourglass dispersion and corresponding edge states remain intact even in the presence of spin-orbit coupling. To support its practical applicability, we have derived various mechanical parameters and analyzed their anisotropic behaviors. We also assessed the material’s response under both compressive and extensive strain conditions, revealing differing trends in energy, size, and hourglass dispersion of the nodal loop. Careful consideration of both the direction and magnitude of strain is essential for specific applications. In conclusion, the hourglass nodal loop state presented in this research offers an ideal foundation for future experimental investigations and explorations.

### Computational details

Based on density functional theory (DFT) (Payne et al., 1992), we performed first-principles calculations with the Vienna Ab initio Simulation Package (VASP) (Hafner, 2008), applying the projected augmented wave method (Steiner et al., 2016). The exchange-correlation interactions were described using the Perdew–Burke–Ernzerhof (PBE) functional within the generalized gradient approximation (GGA) framework (Perdew et al., 1996). To prevent interlayer interactions, a vacuum space of 15 Å was incorporated into the crystal structure model. Long-range van der Waals forces were considered by employing the DFT-D2 method (Hafner, 2008). Our computational setup involved a plane wave basis energy cutoff of 500 eV and utilized a 7 × 7 × 1 Monkhorst–Pack k-point mesh for efficient sampling of the first Brillouin zone. In the context of the projector augmented wave (PAW) method (Blöchl, 1994), valence electron configurations were designated as H (1s^1^), S (3s^2^3p^4^) and Li (2s^1^). For structural relaxation and self-consistent calculations, we defined convergence criteria whereby the residual force per atom remained below 1 × 10^−3^ eV/Å and the energy variation per atom fell under 1 × 10^−6^ eV. The *ab initio* molecular dynamics (AIMD) simulation is performed in a 5 × 5×1 supercell for 2 ps at 100–300 K with a canonical ensemble (Bucher et al., 2011). The exploration of topological properties involved constructing maximally localized Wannier functions with the WANNIER90 code (Mostofi et al., 2008; Mostofi et al., 2014), and subsequently calculating projected surface states using the WANNIERTOOLS package (Wu et al., 2018). Mechanical properties were assessed using the stress-strain method (Wang et al., 1995). To streamline the analysis and processing of results, we utilized the VASPKIT high-throughput package (Wang et al., 2021).

## Results and discussions

**FIGURE 1:**
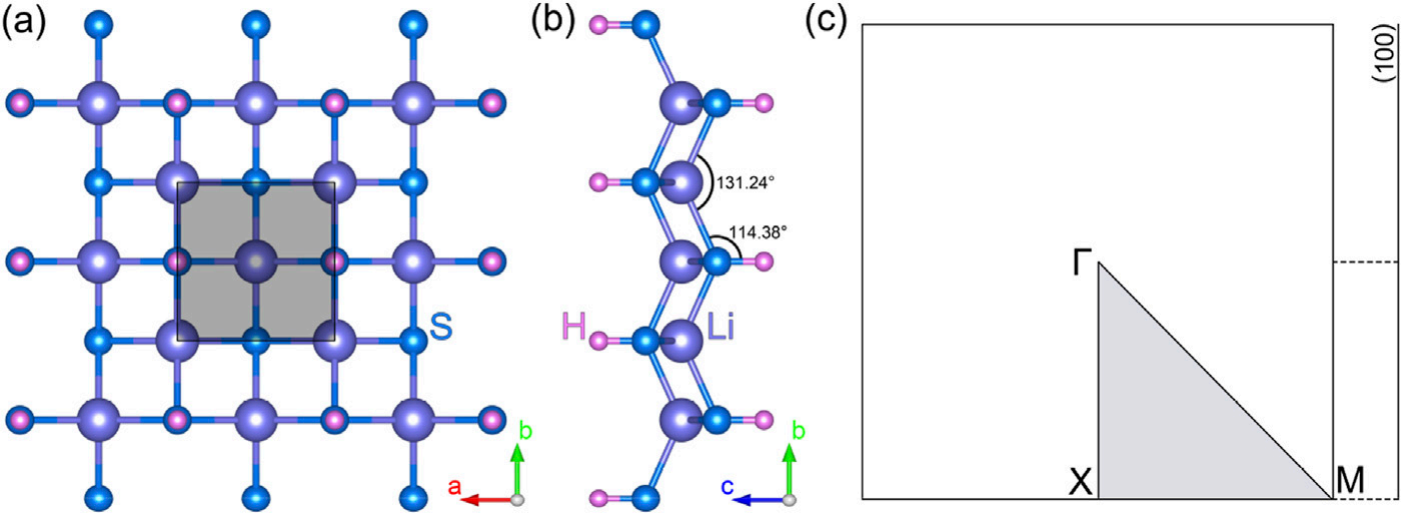
The top **(A)** and side **(B)** views of the monolayer lithium hydrosulfide. The corresponding Brillouin zone **(C)** with high-symmetry points and paths.

The monolayer lithium hydrosulfide (LiHS) features a two-dimensional tetragonal lattice, categorized under the space group P4/nm, No. 129. Figures 1A, B provide the top and side views of the lattice structure, respectively. In the top view, the gray-shaded region represents the primitive cell of the LiHS monolayer, which comprises two hydrogen atoms, two lithium atoms, and two sulfur atoms positioned at the 2a, 2b, and 2c Wyckoff sites, respectively. From the side view, the LiHS lattice reveals a quintuple atomic layer arranged in the sequence H–S–Li–S–H. The lattice constant for the LiHS monolayer is optimized to a = b = 4.515 Å. The bond lengths are measured at 2.478 Å for Li–S and 1.345 Å for S–H. Additionally, the angle of the S–Li–S bond is 131.24°, slightly larger than the Li–S–H bond angle of 114.38°, as illustrated in Figure 1B. Initially reported in the C2DB database (Gjerding et al., 2021; Haastrup et al., 2018), the structure of the LiHS monolayer is noted for its high kinetic stability and potential for exfoliation from its three-dimensional crystalline form. To further assess its thermal stability, we conducted *ab initio* molecular dynamics (AIMD) simulations of the LiHS monolayer. In these simulations, a 5 × 5 × 1 supercell was used, with temperatures set at 100 K, 200 K, and 300 K, respectively. The simulations spanned 2000 steps, with each step representing 1 femtosecond. As demonstrated in Supplementary Figures S1–S3, the total energy of the LiHS monolayer exhibits minor fluctuations over time, maintaining its structural integrity with negligible deformations across all tested temperatures. This confirms the LiHS monolayer’s robust thermal stability, up to temperatures of 300 K. Collectively, these findings of both kinetic and thermal stability underscore the feasibility of experimentally synthesizing the LiHS monolayer, paving the way for its potential applications.

**FIGURE 2:**
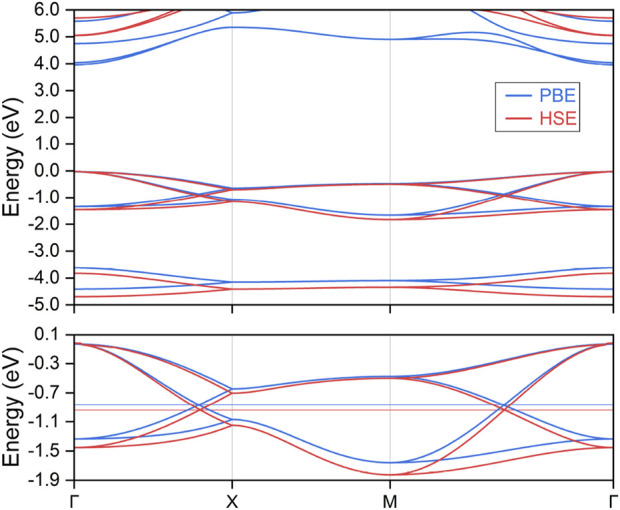
The calculated electronic band structure for the monolayer lithium hydrosulfide under both PBE and HSE formalism calculations. The top valence bands are further enlarged in the bottom panel.

Based on the optimized crystal structure, the electronic band structures of the LiHS monolayer were calculated, with the results presented in Figure 2. The Fermi energy level serves as the reference point, set at 0 eV. High-symmetry k-paths were determined according to the structural crystallographic data and they were selected via the Seek-Path code, as depicted in Figure 1B. It should be noted that spin-orbit coupling was not considered in this analysis, given the light nature of the constituent elements; its effects will be discussed in subsequent sections. The electronic band structures were evaluated using both the PBE and HSE06 formalisms, and these results are combined in the top panel of Figure 2. Analysis shows that the LiHS monolayer exhibits a direct band gap of 3.99 eV with the PBE functional and 5.08 eV with the HSE06 functional, occurring between the valence band maximum and the conduction band minimum at the Γ point. This clearly underscores the insulating nature of the LiHS monolayer. Although the conduction bands shift significantly upwards under the HSE06 formalism, the electronic band dispersion of the top valence bands remains largely similar between the PBE and HSE06 evaluations. This consistency in band dispersion evident with the HSE06 formalism validates the reliability of employing the PBE functional for further analysis. Therefore, PBE will be used for subsequent studies due to its computational efficiency and reliable depiction of band structure trends.

To provide a clearer visualization of the top valence bands, the local band structure is magnified in the bottom panel of Figure 2, with each of the four involved bands highlighted in distinct colors. This enlarged view reveals several instances of band convergence and overlap, notably featuring two prominent hourglass crossing points along the Γ–X and M–Γ paths. These hourglass crossings occur within the same set of valence bands, which are situated close to the Fermi energy level and are distinctly separated from the other bands. This separation simplifies both theoretical analysis and experimental validation. The presence of hourglass band crossings along the Γ–X and M–Γ paths suggests that these crossings may not be isolated phenomena (Yu et al., 2023). Instead, they likely form part of a larger structure known as a nodal line. The LiHS monolayer, with its tetragonal P4/nm space group, exhibits glide mirror symmetry ${Μ}_{z}$ and time-reversal symmetry Τ in the absence of spin orbit coupling (SOC) effect. Under these symmetry operations, the double-degenerate state at the Γ point shares identical eigenvalues of m_z_ (±1), whereas at the X (or M) point, the double-degenerate state exhibits opposite eigenvalues of m_z_ (±i). Considering the band structure along the Γ-X path, as illustrated in Supplementary Figure S4, the eigenvalues associated with m_z_ reveal a partner-switching behavior between the two doublets, leading to a band switch from Γ to X and a Weyl crossing point along this path. Moreover, this band switching is not limited to these paths alone; it occurs along any path, forming an hourglass nodal loop. Such an hourglass nodal loop is uncommon and has been predominantly studied in three-dimensional materials. It holds potential for generating exotic quantum phenomena, such as unconventional superconductivity, non-Fermi liquid behaviors, and fractional quantum Hall states. In contrast to previously reported studies, the current nodal loop in the LiHS monolayer is characterized by a four-band structure, representing one of the most straightforward and simplest configurations.

**FIGURE 3:**
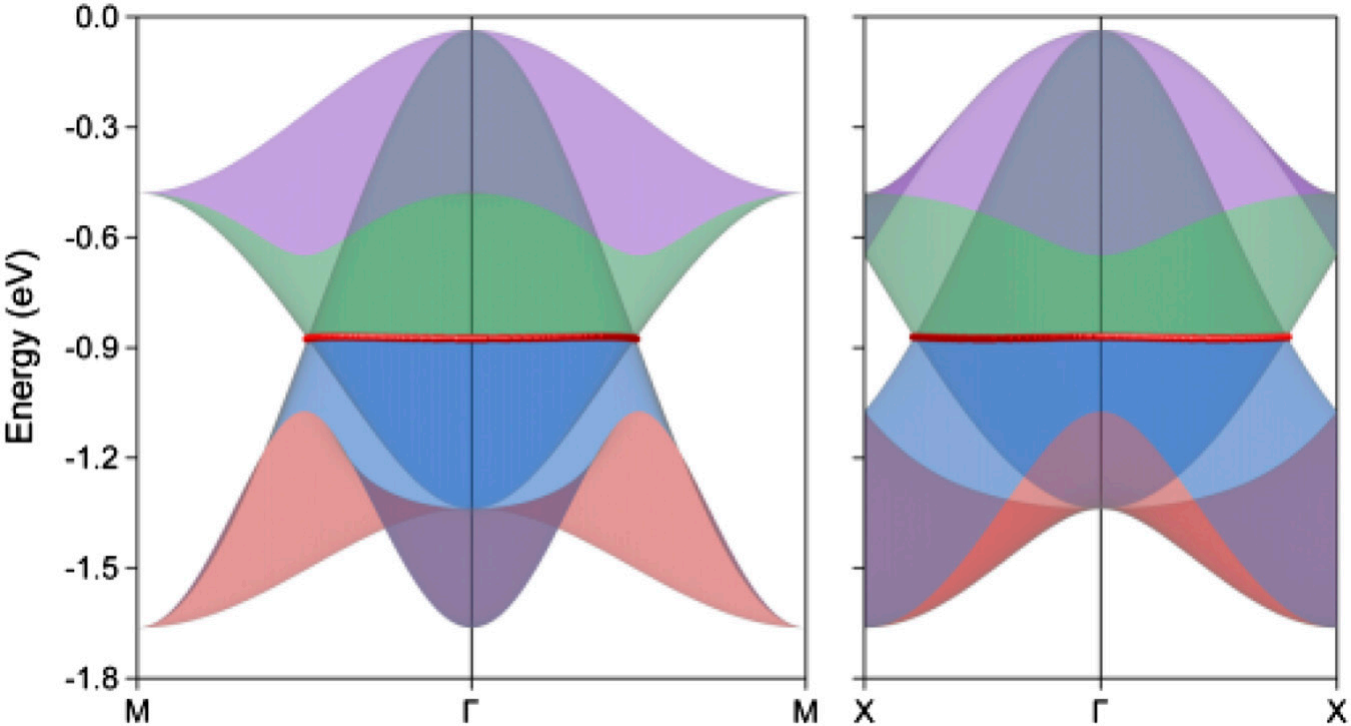
The projection of the three-dimensional band dispersions along M-Γ-M and X-Γ-X paths for the monolayer lithium hydrosulfide. The red spheres correspond to the hourglass crossing points.

Typically, a nodal loop often exhibits finite energy variation and can display different dispersion types or crossing conditions throughout its path. However, it is particularly noteworthy that the two neck points along the Γ–X and M–Γ paths lie at the same energy level, as indicated by the horizontal line in the bottom panel of Figure 2. The complexities inherent in the energy variation and dispersion conditions along the entire hourglass nodal loop make it impossible to determine these aspects through symmetry analysis alone, especially at the critical neck points. Consequently, we conducted a comprehensive band structure analysis across the entire k_z_ = 0 plane, leading to the three-dimensional band dispersions showcased in Figure 3. For this analysis, we selected two projection paths, M–Γ–M and X–Γ–X, and applied consistent color coding to the band surfaces, which align with the local band structures depicted in the bottom panel of Figure 2. We highlighted the hourglass crossing points with red spheres for clarity. These visual representations conclusively demonstrate that the crossing points form a closed nodal loop, thus confirming our earlier symmetry analysis. Importantly, we observed no energy variation at the neck points along this loop. As far as we know, this discovery of a flat nodal loop, characterized in both energy dispersion and spatial distribution, is unprecedented in prior studies and represents a novel finding, particularly within two-dimensional systems. Furthermore, to better visualize band distribution and the configuration of this hourglass nodal loop, we have included the corresponding three dimensional band surface distribution in Supplementary Figure S5 and the hourglass nodal line profile within the k_z_ = 0 plane in Supplementary Figure S6. The nodal loop reveals a slightly distorted circular shape, characterized by a substantially large spatial occupation. Furthermore, we conducted a detailed band segment scan, with the resulting band structures presented in Supplementary Figure S7. Notably, the entire nodal loop exhibits a consistent type-I crossing condition, and the hourglass dispersion becomes increasingly pronounced as one moves from the Γ–X direction to the Γ–M direction. The large dimensions of the nodal loop, combined with the enhanced hourglass dispersion, suggest significant potential for experimental characterization and practical applications, marking it as a compelling subject for further research.

**FIGURE 4:**
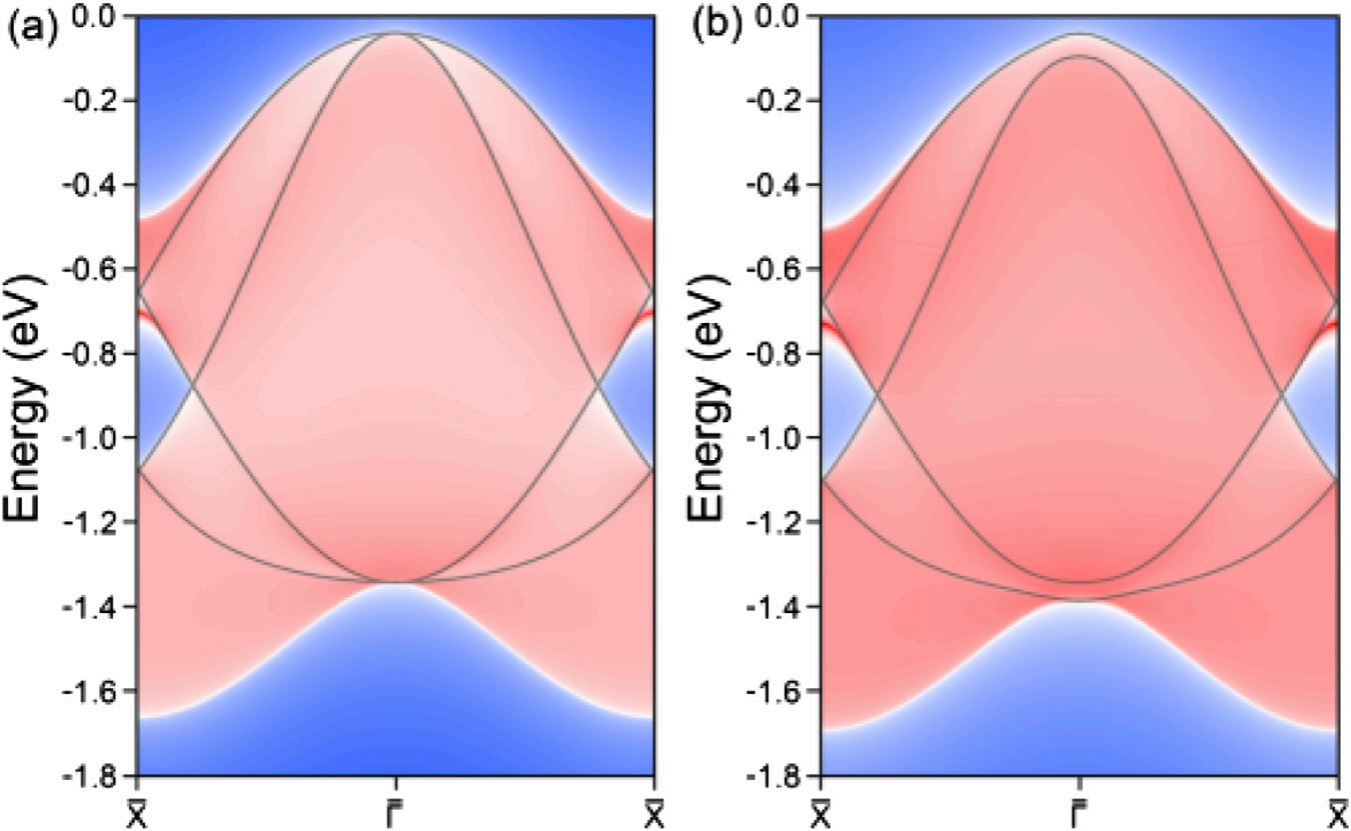
The calculated edge states along the (100) direction for the monolayer lithium hydrosulfide without **(A)** and with **(B)** the spin orbital coupling effect. The bulk band structures are overlaid, showing a strong correspondence, especially in the topological band crossing regions.

In three-dimensional materials, the presence of a topological phase is typically associated with nontrivial surface states. When transitioning to two-dimensional materials, these surface states are scaled down to edge states. For the top four valence bands associated with the hourglass nodal loop in the LiHS monolayer, we performed a detailed decomposition of their orbital contributions, with the results presented in Supplementary Figure S8. Analysis reveals that these four valence bands predominantly consist of p orbitals from S element, particularly the p_x_ and p_y_ orbitals. Moreover, we observe a band inversion feature near the hourglass crossing regions, indicating the potential nontrivial band topology in the LiHS monolayer. Building on these orbital compositions, we successfully constructed a maximally localized Wannier tight-binding Hamiltonian, enabling us to examine the correlated topological edge states. The calculated edge states along the (100) direction of the LiHS monolayer are illustrated in Figure 4. Notably, the bulk band structures are overlaid on the edge projections, showing a strong correspondence, especially in the regions of topological band crossing. As shown in the figure, distinct edge states emerge from the nodal loop crossing points and extend toward the Brillouin zone boundary. Given the substantial size of the nodal loop discussed earlier, the edge states exhibit a relatively limited spatial distribution. Nevertheless, they remain well-separated from the bulk band projection, which enhances their experimental detectability and feasibility. Additionally, we explored the edge states under the influence of SOC effect. Our findings indicate that the band crossing points at the Γ position are opened up by the introduction of a band gap; however, the integrity of the hourglass crossing is preserved, and the corresponding edge states are retained. This robust hourglass nodal loop under SOC presents exciting and attracting possibilities for further research and potential applications.

**FIGURE 5:**
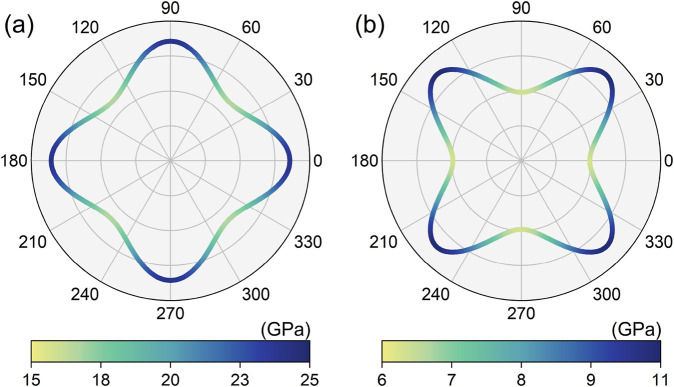
The calculated directional-dependent Young’s modulus **(A)** and shear modulus **(B)** for the monolayer lithium hydrosulfide.

To facilitate future experimental characterization and guide potential applications, we performed a comprehensive evaluation of the mechanical properties of the LiHS monolayer. Several important mechanical parameters are derived, including the three independent elastic constants (C_11_, C_12_, and C_66_), along with Young’s modulus, shear modulus, and Poisson’s ratio, and their values are presented in Table 1. According to the elastic stability criteria for tetragonal structures—namely, C_11_ > 0, C_66_ > 0, and C_11_ > |C_12_|—the LiHS monolayer is confirmed to be mechanically stable. This, combined with other stability factors, indicates a strong potential for the experimental fabrication of the LiHS monolayer. We also investigated the mechanical anisotropy by analyzing the directional dependence of these properties. As illustrated in Figure 5, the LiHS monolayer exhibits relatively large anisotropy in both Young’s modulus and shear modulus, with calculated values of 1.437 and 1.760, respectively. Notably, Young’s modulus exhibits its maximum value along the (100) direction, while the shear modulus reaches its peak along the (110) direction. These anisotropic mechanical characteristics underscore the importance of directional dependence, which is especially relevant for applications that require tailored structural integration or the formation of heterojunctions. Given the pronounced anisotropic nature of the LiHS monolayer, it is essential to consider careful selection of directions in various engineering and technological applications. This insight will enable more effective utilization of the material’s mechanical properties in future designs and integrations.

**TABLE 1:** The calculated various elastic constants (*C*_11_, *C*_12_, and *C*_66_), Young’s modulus (*E*), shear modulus (*G*), and Poisson’s ratio (*ν*) for the monolayer lithium hydrosulfide.

*C*_11_	*C*_12_	*C*_66_	*E*	*G*	*ν*
(N/M)					
24.581	3.815	5.898	20.329	8.141	0.2841

**FIGURE 6:**
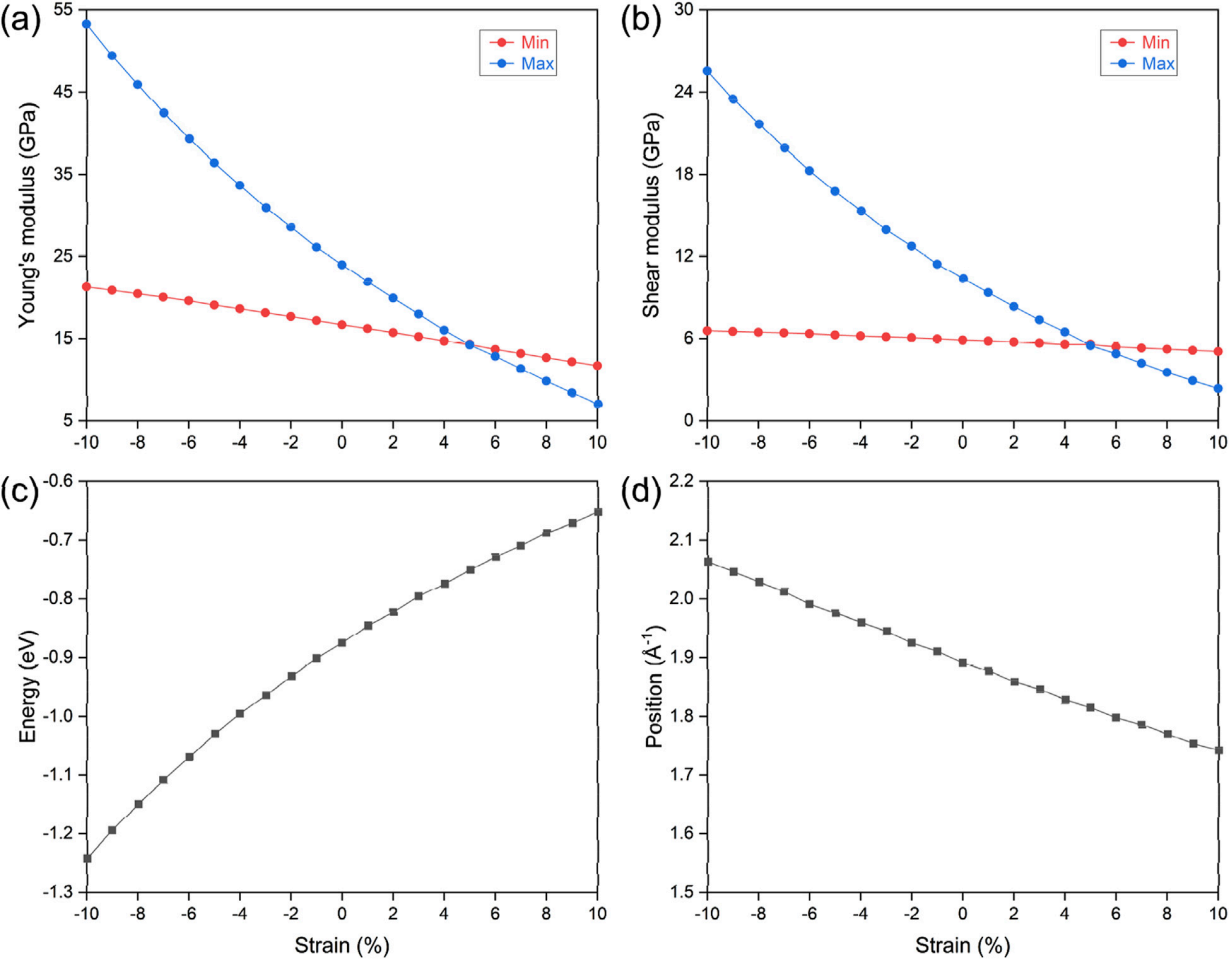
The strain effect on the monolayer lithium hydrosulfide. The variation of the maximum and minimum values of the Young’s modulus **(A)** and shear modulus **(B)** of the monolayer lithium hydrosulfide, and the energy level **(C)** and space position **(D)** of the hourglass nodal loop state.

Beyond the mechanical properties pertinent for structural integration, it is imperative to consider the effects of strain during both experimental preparation and practical application. Lattice variation is pivotal in determining and influencing the physical properties of materials, especially in two-dimensional systems where in-plane strain occurs during substrate-supported preparation. Moreover, strain significantly affects electronic topological properties, notably altering the winding configuration of nodal loops and impacting the band crossing conditions along these loops. Here, we examine the influence of uniform in-plane strain on the electronic topological and mechanical properties of the LiHS monolayer. The results are summarized in Figure 6, with the strain range considered from −10% to +10%. The maximum and minimum values of Young’s modulus, as shown in Figure 6A, are defined based on their spatial distribution, with the maximum occurring along the (100) direction and the minimum along the (110) direction. In Figure 6B, the maximum value of the shear modulus is located along (110) direction and the minimum value along (100) direction. As strain is varied from compressive to extensive, corresponding to range from −10% to +10%, both Young’s modulus and shear modulus decrease monotonically, with the maximum values exhibiting a more rapid change than the minimum values. At a strain of +5%, we note a significant curve crossing, where the maximum and minimum values converge, indicating a shift toward isotropic behavior for both Young’s modulus and shear modulus. The directional distribution at this strain point is illustrated in Supplementary Figure S9, revealing an almost perfectly circular shape that further confirms this isotropy. Regarding the hourglass nodal loop, we assessed the energy level and spatial position of the neck point along the Γ–M path, with their variations under strain conditions displayed in Figures 6C, D, respectively. Specifically, the energy of the hourglass nodal loop shifts effectively toward the Fermi level as strain transitions from compressive to extensive; however, the size of the nodal loop decreases under the same stress conditions.

**FIGURE 7:**
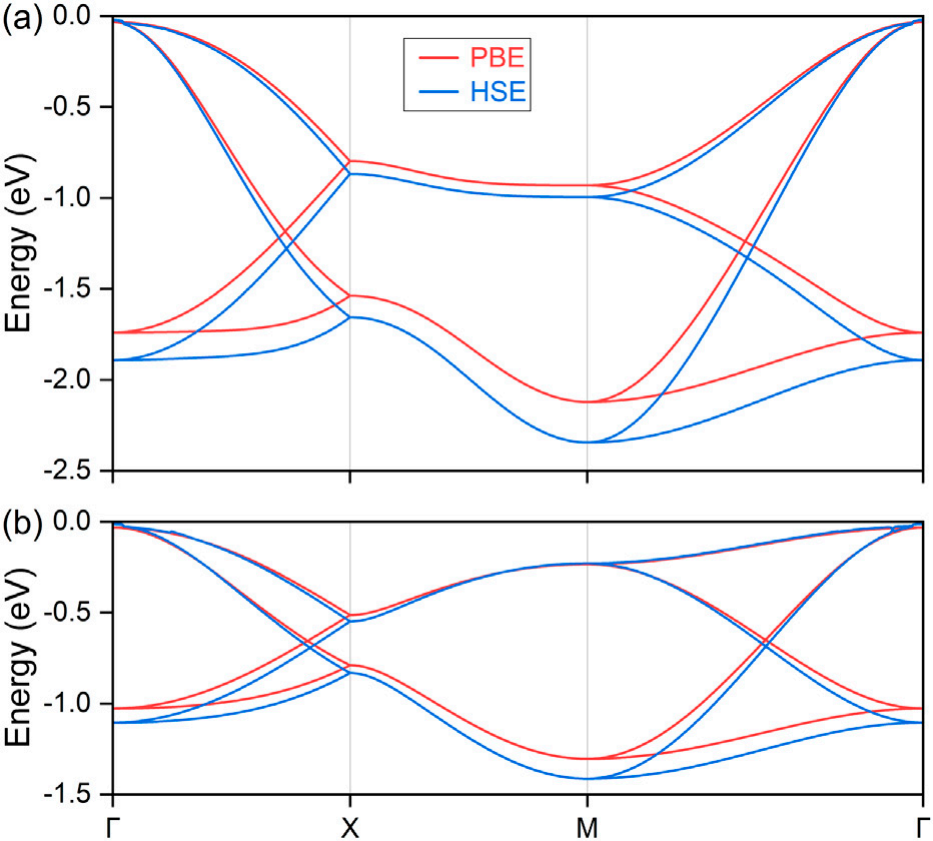
The calculated electronic band structure for the monolayer lithium hydrosulfide under −10% compressive strain **(A)** and +10% extensive strain **(B)**.

To further confirm the hourglass dispersion of the nodal loop under strain conditions, we present the local band structures of the top four valence bands. These are shown under −10% compressive strain in the top panel of Figure 7 and +10% extensive strain in the bottom panel. Both the PBE and HSE06 methods are considered, and their results show good consistence. Notably, the same energy scale ratio is applied across different strains. It is evident that the hourglass dispersion is significantly contracted in energy scale under extensive strain. Considering the mechanical properties under strain, the trade-off between energy, size, and hourglass dispersion must be carefully evaluated for specific applications. Consequently, for future experimental investigations and practical applications of the LiHS monolayer, both the direction and magnitude of strain should be carefully considered. These strain characteristics not only highlight the material’s capabilities for detailed analysis but also position it as a promising candidate for various future technological applications.

## Conclusion

In this study, we identify a topological hourglass nodal loop state in the LiHS monolayer through first-principles calculations. This unique nodal loop involves only four bands without interference from other bands, representing the simplest configurations. Using symmetry considerations and an effective Hamiltonian model, we elucidate the formation mechanism of this nodal loop and its hourglass dispersion. Three-dimensional band surface scans confirm the hourglass crossing along the entire nodal loop. Notably, this loop, with its slightly distorted circular shape, is flat in both energy dispersion and spatial distribution and occupies a substantially large spatial area. Orbital decompositions and a tight-binding Hamiltonian reveal edge states along the (100) direction. These states are separated from the bulk band projection and span through the Brillouin zone. Importantly, given the light constituent elements, the hourglass dispersion and corresponding edge states are retained under the spin-orbit coupling effect. This robust hourglass nodal loop enhances the potential for experimental detection and applications. To support practical environmental applications, we have derived key mechanical parameters and examined their anisotropic behaviors, particularly for Young’s modulus and shear modulus. We further evaluate strain conditions under both compressive and extensive stresses, showing different variation tendencies in energy, size, and hourglass dispersion of the nodal loop. Both the direction and magnitude of strain should be carefully considered for specific applications. Overall, the hourglass nodal loop state examined in this study provides an ideal platform for future experimental investigations and explorations.

## Data Availability

The raw data supporting the conclusions of this article will be made available by the authors, without undue reservation.
