# Supplementary material for: Ideal hourglass nodal loop state in the monolayer lithium hydrosulfide
Source: Front Chem. 2024 Dec 2;12:1500989. doi: 10.3389/fchem.2024.1500989 (PMC11650700; doi:10.3389/fchem.2024.1500989)
Supplement: Supplementary file 1 [file DataSheet1.docx]

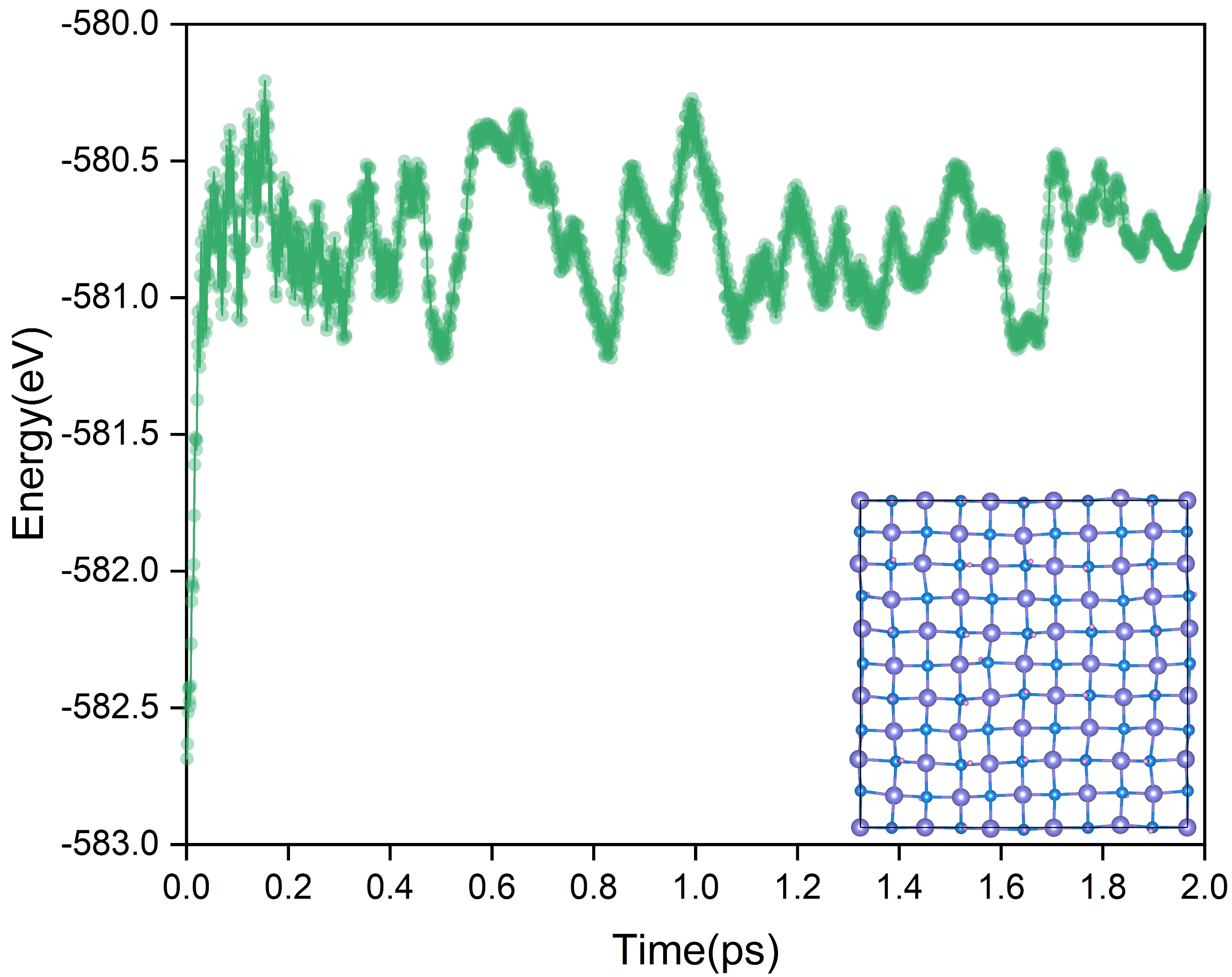


Figure S1: The total energy fluctuations of the monolayer lithium hydrosulfide with time revolution under the AIMD simulations at 100K. The 5×5×1 supercell is applied and the final structure after simulation is shown as the inset, which only exhibits slight structural variation.


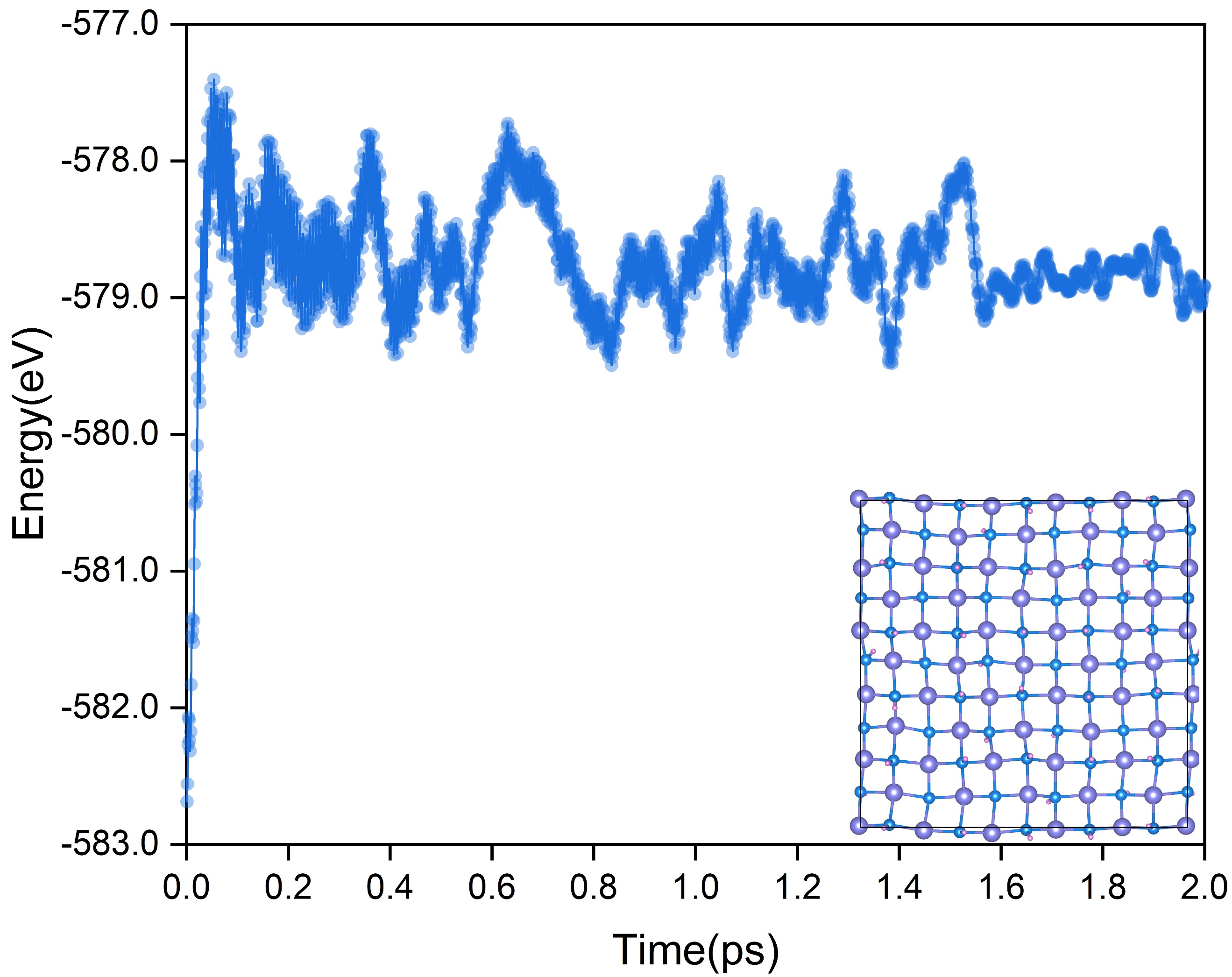


Figure S2: The total energy fluctuations of the monolayer lithium hydrosulfide with time revolution under the AIMD simulations at 200K. The 5×5×1 supercell is applied and the final structure after simulation is shown as the inset, which only exhibits slight structural variation.


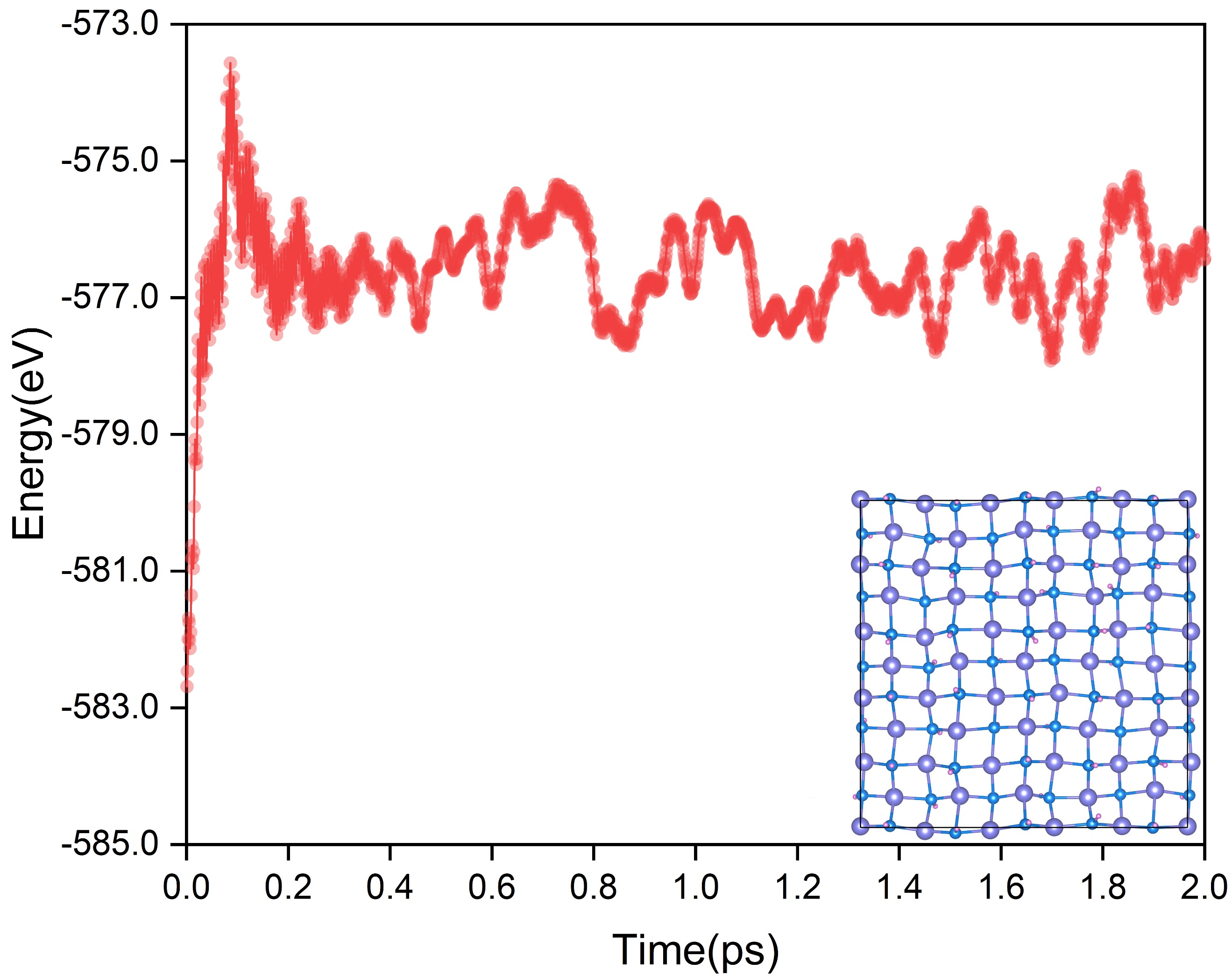


Figure S3: The total energy fluctuations of the monolayer lithium hydrosulfide with time revolution under the AIMD simulations at 300K. The 5×5×1 supercell is applied and the final structure after simulation is shown as the inset, which only exhibits slight structural variation.


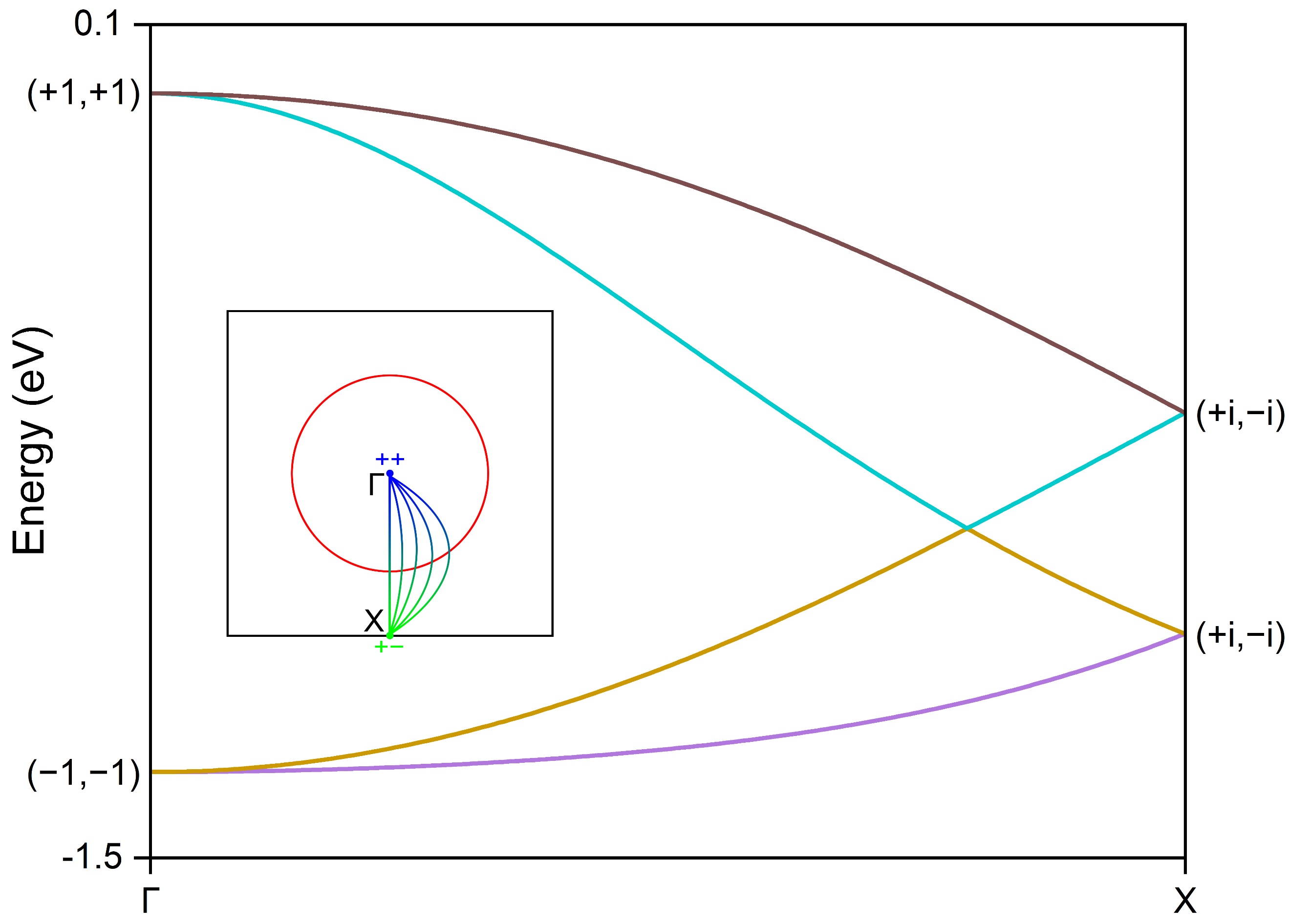


Figure S4: Schematic illustration for the hourglass dispersion along Γ-X path. The labels indicate the $\text{M}_{\text{z}}$ eigenvalues. Partner switching between two doublets leads to the twofold Weyl crossing point within the Γ-X path.


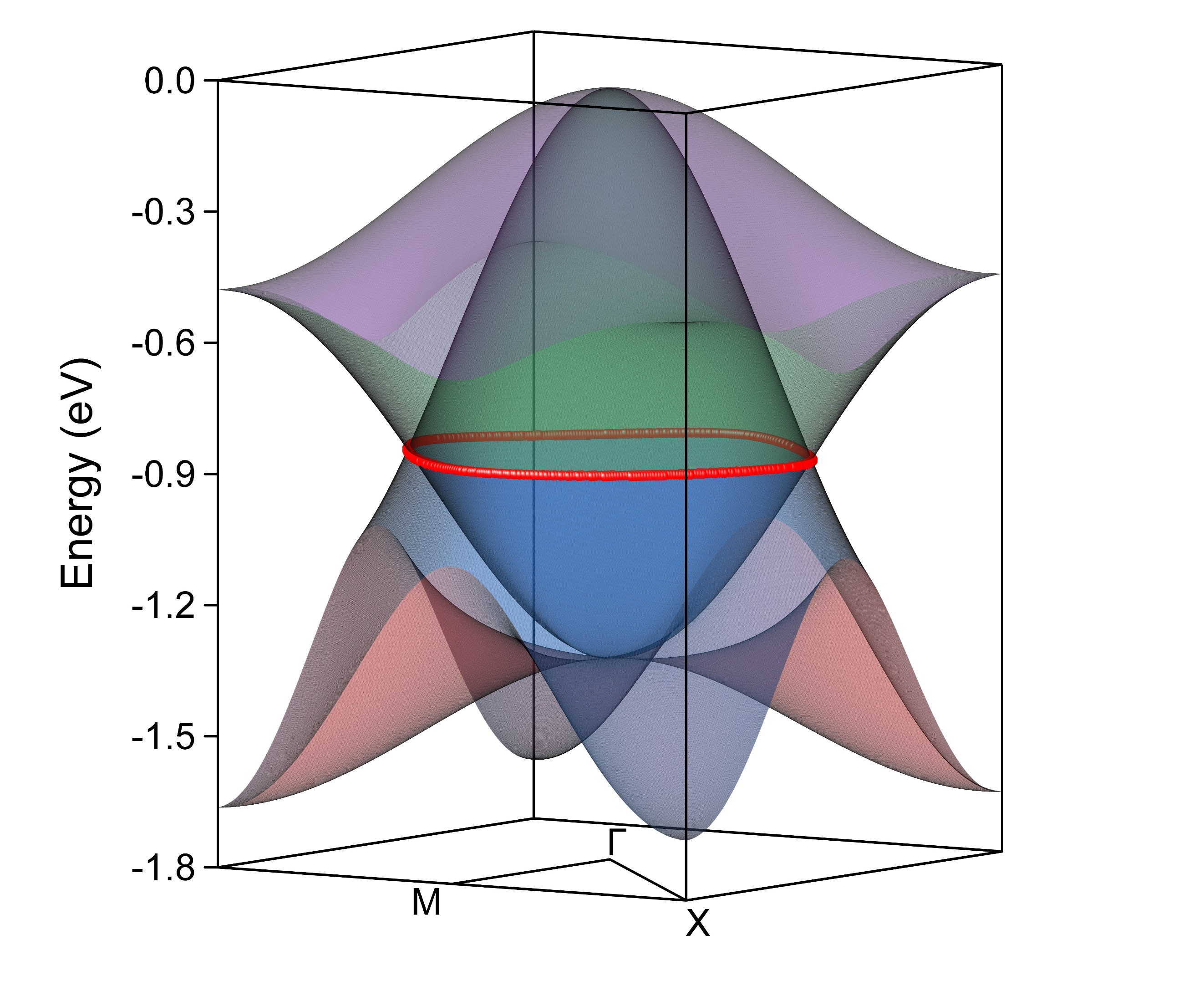


Figure S5: Schematic illustration for the hourglass dispersion along Γ-X path. The labels indicate the $\text{M}_{\text{z}}$ eigenvalues. Partner switching between two doublets leads to the twofold Weyl crossing point within the Γ-X path.


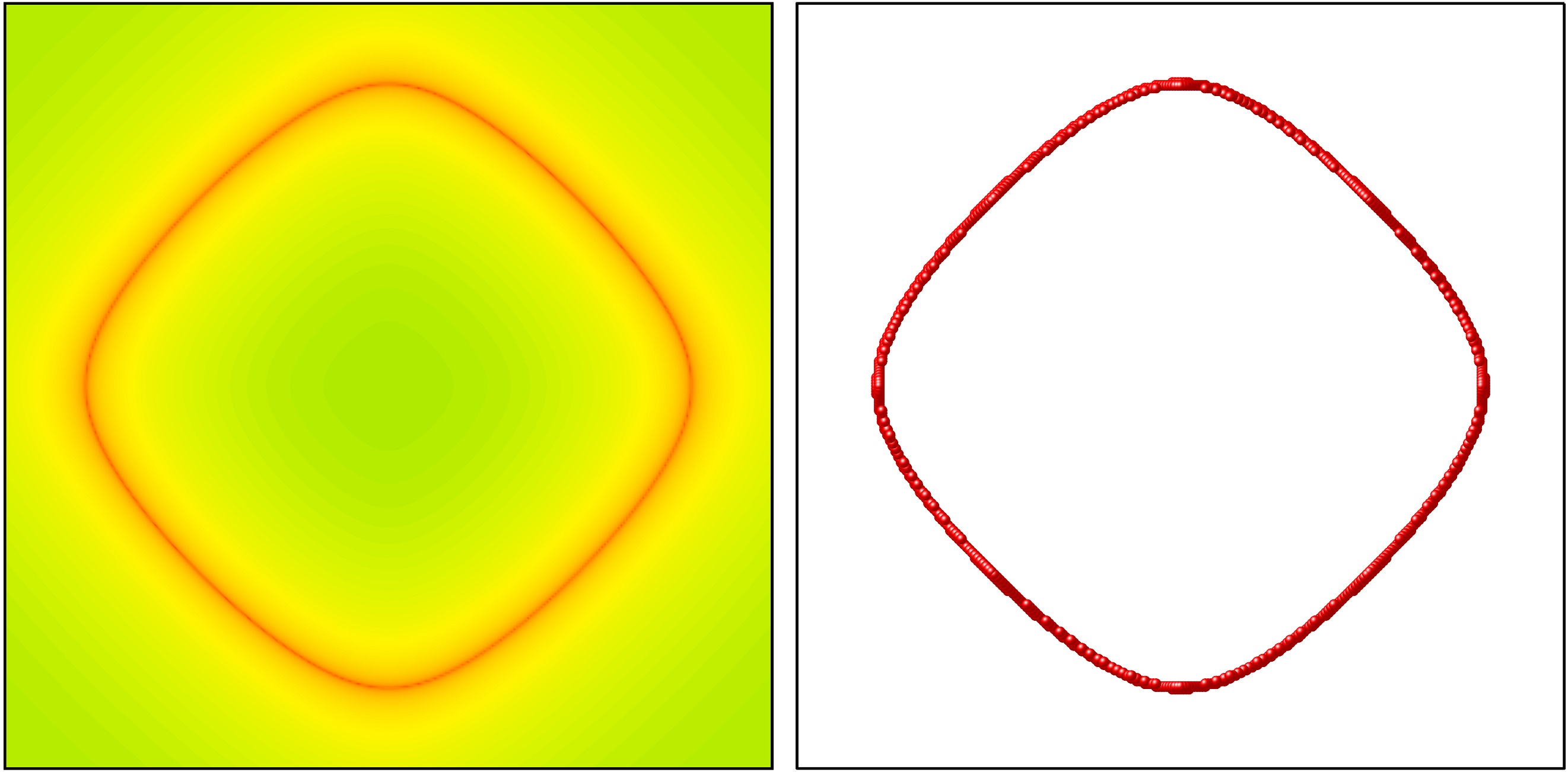


Figure S6: The profile of the hourglass nodal loop in the k_z_ = 0 plane. The left panel shows the energy difference for the two crossing bands of the nodal loop and the right panel shows the crossing points of the two bands.


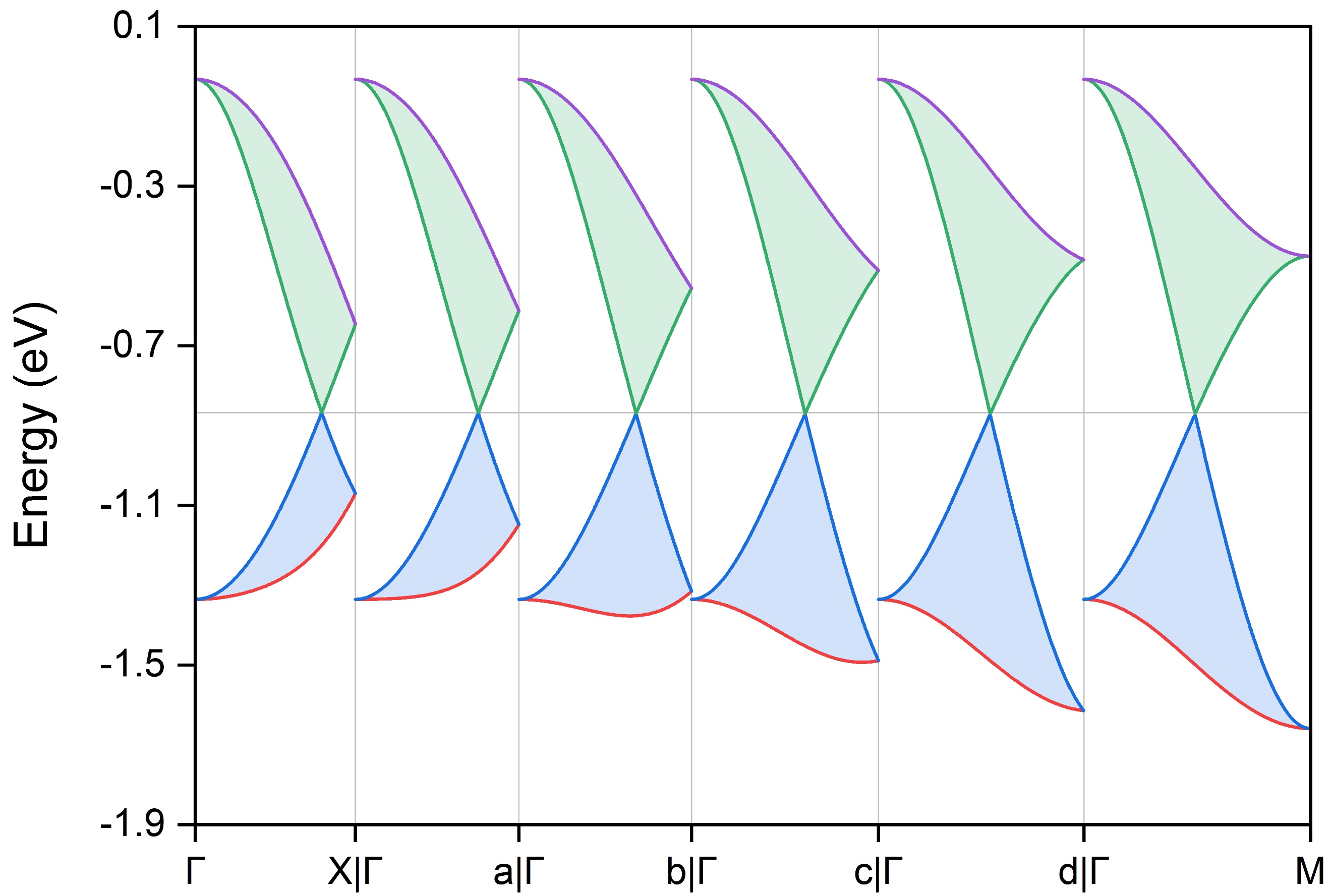


Figure S7: The calculated electronic band structure for the monolayer lithium hydrosulfide under various path segments distributed between X and M. The hourglass dispersion shape is highlighted by the translucent color areas.


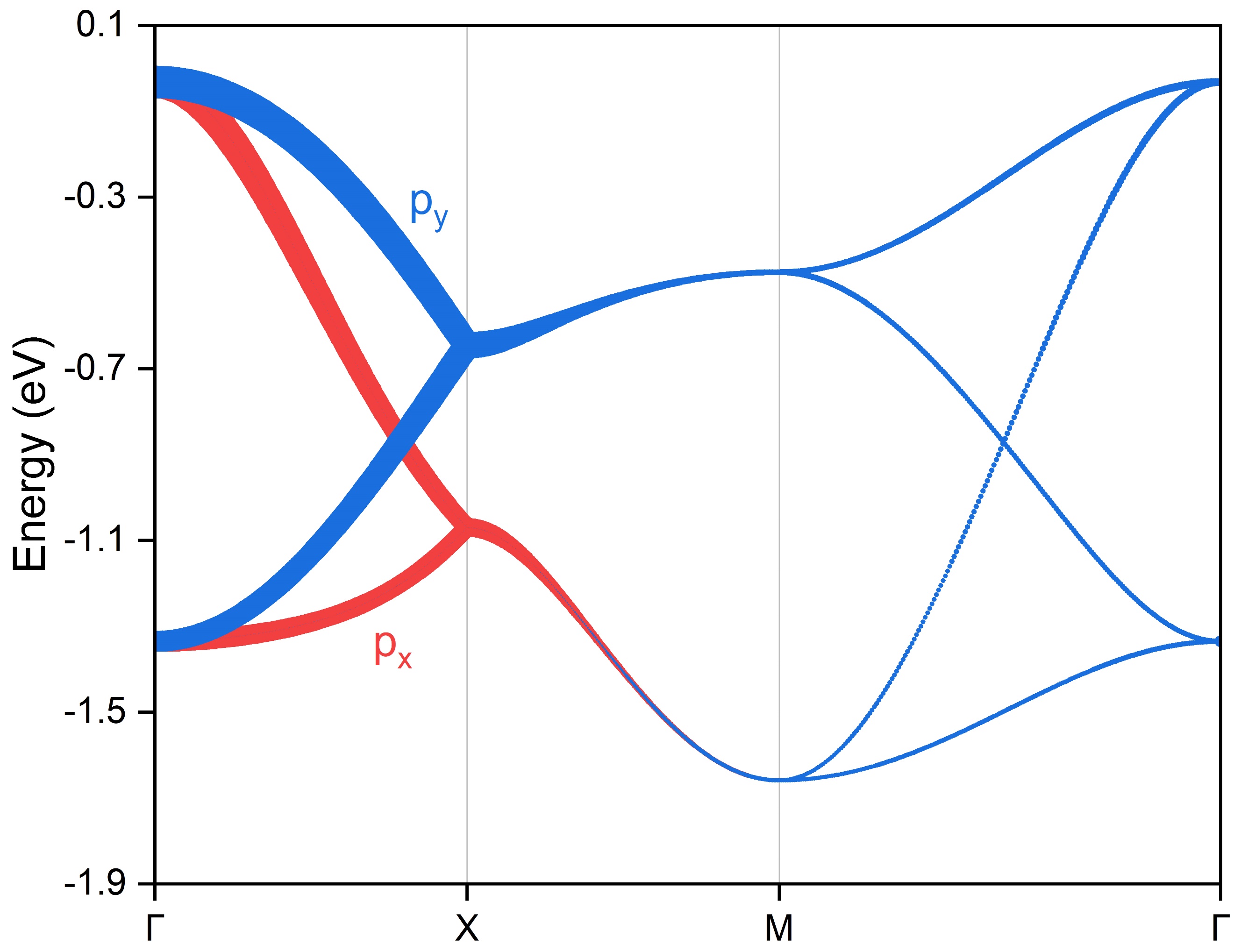


Figure S8: The projected electronic band structures for the monolayer lithium hydrosulfide with the main p_x_ and p_y_ orbital contributions from S element overlaid. The contribution weight is proportional to the line width.


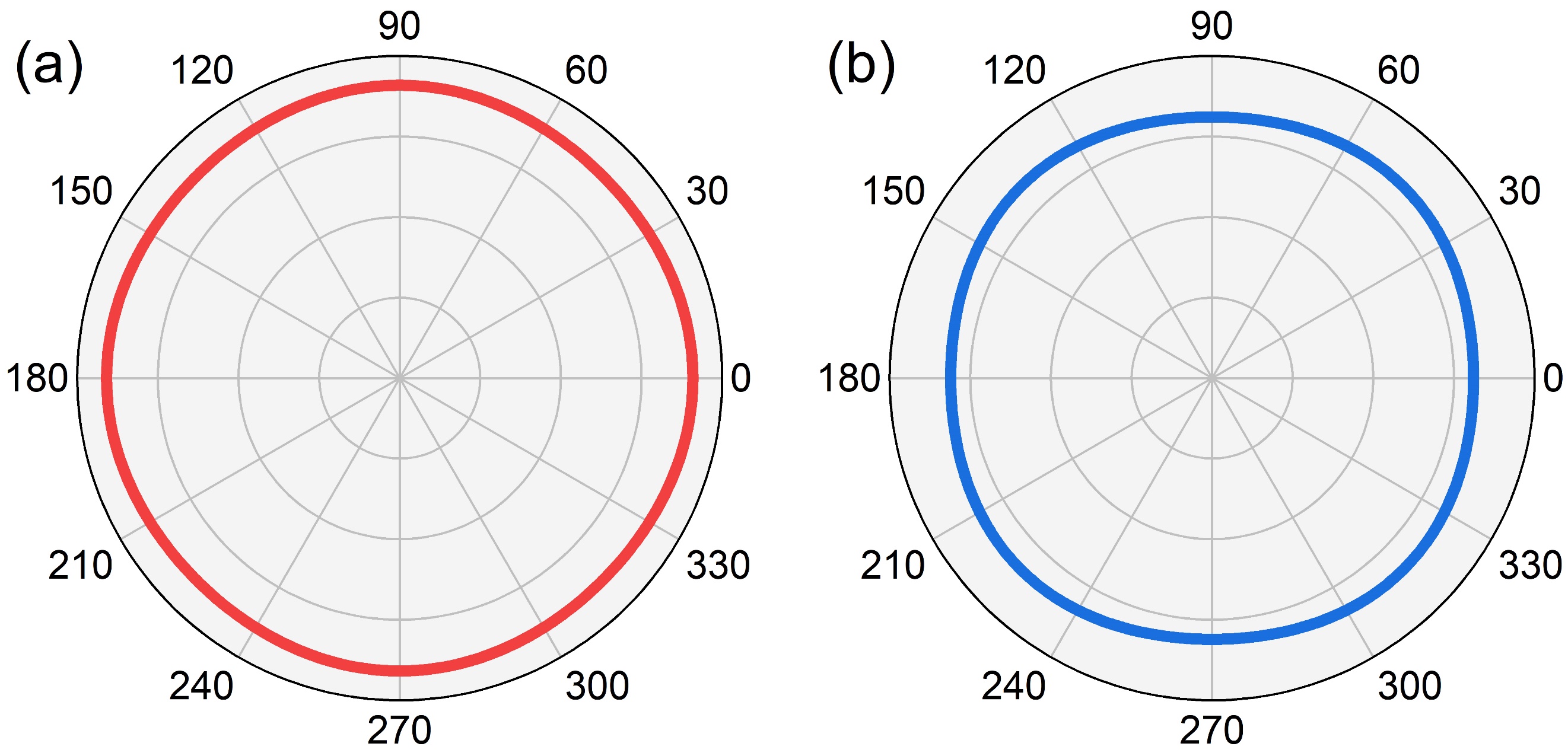


Figure S9: The calculated directional-dependent Young’s modulus (a) and shear modulus (b) for the monolayer lithium hydrosulfide under +5% extensive strain.
